# Citrus psorosis virus 24K protein inhibits the processing of miRNA precursors by interacting with components of the biogenesis machinery

**DOI:** 10.1128/spectrum.03513-23

**Published:** 2024-05-24

**Authors:** Facundo E. Marmisolle, María B. Borniego, Damián A. Cambiagno, Lucia Gonzalo, María L. García, Pablo A. Manavella, Carmen Hernández, Carina A. Reyes

**Affiliations:** 1 Instituto de Biotecnología y Biología Molecular, CCT-La Plata, CONICET-UNLP, La Plata, Buenos Aires, Argentina; 2 Instituto de Agrobiotecnología del Litoral (CONICET-UNL), Cátedra de Biología Celular y Molecular, Facultad de Bioquímica y Ciencias Biológicas, Universidad Nacional del Litoral, Santa Fe, Argentina; 3 Instituto de Biología Molecular y Celular de Plantas, CSIC-UPV, Valencia, Spain; USDA-ARS-NPRL, Dawson, Georgia, USA

**Keywords:** miRNA, biogenesis, machinery, Ophiovirus, *Citrus sinensis*, HYPONASTIC LEAVES 1, SERRATE

## Abstract

Sweet orange (*Citrus sinensis*) is one of the most important fruit crops worldwide. Virus infections in this crop can interfere with cellular processes, causing dramatic economic losses. By performing RT-qPCR analyses, we demonstrated that citrus psorosis virus (CPsV)-infected orange plants exhibited higher levels of unprocessed microRNA (miRNA) precursors than healthy plants. This result correlated with the reported reduction of mature miRNAs species. The protein 24K, the CPsV suppressor of RNA silencing (VSR), interacts with miRNA precursors *in vivo*. Thus, this protein becomes a candidate responsible for the increased accumulation of unprocessed miRNAs. We analyzed 24K RNA-binding and protein-protein interaction domains and described patterns of its subcellular localization. We also showed that 24K colocalizes within nuclear D-bodies with the miRNA biogenesis proteins DICER-LIKE 1 (DCL1), HYPONASTIC LEAVES 1 (HYL1), and SERRATE (SE). According to the results of bimolecular fluorescence complementation and co-immunoprecipitation assays, the 24K protein interacts with HYL1 and SE. Thus, 24K may inhibit miRNA processing in CPsV-infected citrus plants by direct interaction with the miRNA processing complex. This work contributes to the understanding of how a virus can alter the regulatory mechanisms of the host, particularly miRNA biogenesis and function.

**IMPORTANCE:**

Sweet oranges can suffer from disease symptoms induced by virus infections, thus resulting in drastic economic losses. In sweet orange plants, CPsV alters the accumulation of some precursors from the regulatory molecules called miRNAs. This alteration leads to a decreased level of mature miRNA species. This misregulation may be due to a direct association of one of the viral proteins (24K) with miRNA precursors. On the other hand, 24K may act with components of the cell miRNA processing machinery through a series of predicted RNA-binding and protein-protein interaction domains.

## INTRODUCTION

In eukaryotes, miRNAs regulate gene expression through RNA silencing at the post-transcriptional level and thus play an essential role in most biological processes, including the responses to biotic and abiotic stresses (1–5). Modern bioinformatic, genetic, biochemical, and molecular approaches have become fundamental tools in the research of regulatory functions of miRNAs in plant-pathogen interactions (6). The biogenesis of miRNAs is a multistep process including transcription, processing, modification, and assembly of the RNA-induced silencing complex (RISC) (7–9).

In plants, RNA polymerase II transcribes *MIRNA* loci to generate primary miRNAs (pri-miRNAs), which are processed first into the pre-miRNA fold-back and then into mature ~21 nt miRNA duplexes, either in the nucleoplasm or coupled to transcription (10–14). The RNase-III endonuclease DCL1, together with the RNA binding protein HYL1 and the zinc finger protein SE, recognizes and processes pri-miRNAs into mature miRNA duplexes (15, 16). In addition, many accessory proteins regulate miRNA biogenesis at different steps (7, 17–19). HYL1 seems to have strong implications in a precise cleavage of miRNAs (20) and interacts with HUA ENHANCER 1 (HEN1) to act as a scaffold and assist miRNA methylation (21–23).

Mature miRNAs are loaded into an ARGONAUTE (AGO) protein to assemble the miRNA RNA-Induced Silencing Complex (miRISC). AGO1 is the main effector of the miRNA pathway in plants (24). Recently, researchers have demonstrated that AGO1 is partially loaded with miRNA duplexes in the nucleus, in a process assisted by HYL1 and CARP9, and then exported to the cytoplasm as an AGO1:miRNA complex (25, 26). The discovery of the nuclear loading of AGO1 not only builds a new model for miRNA export but also reinforces the proposed nuclear functions of AGO1 (27–29). Once in the cytosol, the miRISC complex executes the silencing of endogenous transcripts that share complementary sequences at miRNA-target sites. Silencing occurs via endonucleolytic cleavage, known as “slicing” (30), and/or translational repression, possibly coupled to accelerated mRNA decay (31–34).

Apart from the pool of endogenous miRNAs produced by hosts, viruses also can generate diverse types of small RNAs, which they use to ensure infection (35, 36). On the other hand, plants also deploy a defense battery involving miRNAs, which occur naturally to defend the cells against virus or viroid attacks. For example, miRNA-mediated changes in gene expression modulate viral replication, antiviral immune responses, viral latency, and pathogenesis (3). Researchers have documented numerous cases of altered host miRNA expression in response to plant virus infection. Some of these cases are *Arabidopsis thaliana* [infected with tobacco mosaic virus (TMV) (34); tomato plants infected with cucumber mosaic virus (CMV) and TMV (37); rice stripe virus (RSV) infecting rice (38, 39); grapevine vein clearing virus (GVCV)-infected grapevine (40); and potato virus Y (PVY) isolate PVY^C^ infecting tomato plants (41)].

Although all those studies suggest that miRNAs are involved in host-virus interactions and that, in many cases, this kind of response could be due to the interaction of the viral suppressor of RNA silencing proteins (VSR) with effector components of the silencing mechanism such as AGO proteins, how these pathogens regulate miRNA processing and accumulation remains elusive. Interestingly, the rice stripe virus NS3 protein regulates pri-miRNA processing by assisting the process and increasing the accumulation of mature miRNA species (42).

CPsV is a tripartite, non-enveloped, negative-sense, single-stranded RNA (ssRNA) virus of the Aspiviridae family (formerly Ophioviridae) within the genus Ophiovirus (43). The viral genome is composed of an RNA 1 that encodes a 280 kDa replicase (RdRp) (44) and a 24 kDa protein (24K) that affects miRNA maturation and suppresses RNA silencing (45). RNA 2 encodes a 54 kDa aspartil protease (54K) involved in virus movement (46–48) and also suppresses RNA silencing. RNA 3 encodes the coat protein (CP) of 48 kDa (49, 50).

In a previous study, we had shown that two distantly related CPsV isolates induce a reduction in the accumulation of a set of mature species of endogenous miRNAs in infected *Citrus sinensis* plants by impeding the processing of miRNA precursors (45). We also validated transcript targets of some of these *C. sinensis* miRNAs and evidenced that, concomitantly with the reduction of mature miRNA species, many of these targets accumulated in infected samples compared to healthy controls. On the other hand, the expression of the target genes positively correlates with symptom severity (51). In our previous study, the 24K protein (but not the 54K protein) interacted with miRNA precursors *in vivo* (45).

In the present research, we analyzed the accumulation of miRNA precursors in CPsV-infected citrus plants in relation to healthy plants. The study included the assessment of subcellular localization patterns of 24K alone or colocalizing with different components of the miRNA biogenesis machinery (DCL1, HYL1, or SE). Molecular interactions between 24K and DCL1, HYL1, or SE were analyzed by two different methodologies: bimolecular fluorescence complementation and co-immunoprecipitation. Bioinformatic analyses of 24K were also performed to find relevant regions or domains for its interaction with miRNA biogenesis components or with miRNA precursors.

## MATERIALS AND METHODS

### Plant citrus material and CPsV isolates

CPsV isolates used in this study were the Argentine CPsV 90-1-1 (INTA, Concordia, Argentina) (52) and CPV4 from Florida, USA (53), but probably of Texas origin (54). Pineapple sweet orange plants [*C. sinensis* (L.) Osbeck], which were provided by INTA EEA-Concordia, were infected in the stem through graft inoculation by using a small chip taken from infected bark or healthy tissue for healthy controls (55). Leave tissues of *C. sinensis* infected with CPsV were collected before complete necrosis (shock symptom) became apparent. Equivalent material from healthy plants and infected tissue expressing chlorosis symptoms (flecking) were also collected.

Agro-infiltration experiments were performed in epidermal cells of 5- to 6-week-old *Nicotiana benthamiana* (*N. benthamiana*) plants maintained in a growth chamber at 23–25°C with a 16 h light/8 h dark photoperiod.

### RNA isolation and cDNA preparation

Total RNA was isolated from 50 to 200 mg of tissue previously ground in liquid nitrogen by using TriReagent (Molecular Research Center, Inc., Cincinnati, OH, USA) and following the manufacturer’s instructions. The RNA integrity was assessed by 1% agarose gel electrophoresis. The total RNA was processed with RQ1 Rnase-free Dnase (Promega) for 60 min at 37°C to eliminate potential DNA contamination. An aliquot of the treated RNA samples (about 1.5 µg) was used to prepare cDNA using M-MLV reverse transcriptase (Promega) and specific primers (Table 1) in the presence of Native Rnasin Ribonuclease Inhibitor (Promega).

**TABLE 1:** Oligonucleotide sequences used for pre-miRNA quantification by qPCR in *Citrus sinensis* leaves^*a*^

Oligonucleotide	Sequence 5′ → 3′
pre156Adir	TGACAGAAGAGAGTGAGCAC
pre156Arev	GCTGACAGAAAGAGCAGTGA
pre167Ddir	CACACAAGCAGTCTACAAGG
pre167Drev	CCGCAAGTAGGAAGGAGTGA
pre169Ddir	AATATAATATCATTGTTTGTTAGCC
pre169Drev	CTGTGACTTAGCCAAGGAGACTGCC
pre171Adir	AACGGAGATGTTGGAACGGC
pre171Arev	GAGATATTGGCACGGCTCAA
pre172Adir	CTGTAGCAGCGTCCTCAAGA
pre172Arev	CCGTTGCAGCATCATCAAGA
pre393Bdir	CTTGATTAGTGCAGGTGGAGAG
pre393Brev	ATTTAGAGCCATAGATGGGG

^*a*^ A, B, and D refers to individual members of the pre-miRNA families.

### Detection of miRNA precursors by reverse transcription and quantitative polymerase chain reaction

Reverse transcription and quantitative polymerase chain reaction (RT-qPCR) was performed to analyze miRNA precursors from the cDNAs (see RNA isolation and cDNA preparation) as templates for qPCRs. The absence of contaminant genomic DNA was confirmed in Dnase-treated RNA samples. The qPCRs were performed using a qTOWER 2.0 (Analytik Jena AG) and 5× HOT FIREPol EvaGreen qPCR Mix (Rox) (Solis BioDyne) following the manufacturer’s instructions. The reaction conditions were as follows: 95°C for 10 min, then 60 cycles of 20 s at 95°C, 30 s at 48–55°C, and 20 s at 72°C, followed by a melting curve at 60–95°C 6 s with ΔT 1°C. The primer sequences are indicated in Table 1.

A unique product of the expected size was verified on ethidium bromide-stained agarose gels. Actin (Cs6g06250) and ubiquitin (Cs6g04450) amplifications were used as internal controls (56). All RT-qPCR experiments consisted of at least three biological and three technical replicates.

The primers to detect *C. sinensis* miRNA precursor genes and reference genes were designed using citrus sequences deposited in miRBase (https://www.mirbase.org/) and Phytozome v12 (https://phytozome.jgi.doe.gov/pz/portal.html) and in the Citrus Pan-genome to Breeding Database (http://citrus.hzau.edu.cn/index.php).

### Statistical analysis

The geometric Ct mean of two reference genes (actin and ubiquitin) and efficiency average were employed for normalizing the RT-qPCR data. The relative expression was indicated as *E*^−Δcttarget^/*E*^−Δcthousekeeping^, where *E* corresponds to the primer efficiency value. Analysis of variance (ANOVA) with a significance level of 5.0% (*P* < 0.05) followed by Tukey post hoc test was used for mean comparisons. Means with a common letter are not significantly different (*P* > 0.05). The assumptions of normality and homogeneity of variance were checked before performing every parametric analysis.

### Plasmid constructs

The 24K open reading frame (ORF) without a stop codon was cloned into pCR8/GW/TOPO (Invitrogen, Carlsbad, CA, USA.) to express the 24K:mRFP fusion protein (pB7RWG2-24K). The resulting entry plasmid pCR8:24K was digested with XhoI and recombined with destination vector pB7RWG2 (57) using LR clonase mix (Invitrogen), according to the manufacturer’s instructions. The correct cloning and insert orientation were confirmed by sequencing.

Different 24K mutants were generated using overlapping PCR from pCR8:24K. pCR8:24ΔN: first 33 amino acids from N-terminus were deleted. pCR8:24ΔC: last 17 C-terminal amino acids were deleted. pCR8:24ΔNES: amino acids between positions 156 and 170 were deleted. Finally, pCR8:24 W^15^A: Tryptophan 15, which is part of a WG motif, was replaced by Alanine.

The coding sequence of 24K from pCR8:24K was cloned in N/C-mCitrine adapted for bimolecular fluorescence complementation (BiFC) compatible pGreen destination vectors (19, 58) using LR clonase mix (Invitrogen), according to the manufacturer’s instructions. Same strategy was used to clone 24K mutant versions. These vectors have the N-terminal and C-terminal regions of the fluorescent protein mCitrine, respectively. The correct cloning and insert orientation were confirmed by sequencing.

The verified constructions were transferred to the *Agrobacterium tumefaciens* strain GV3101 by electroporation.

### Transient expression and confocal detection

*A. tumefaciens* infiltration of *N. benthamiana* leaves was performed to conduct subcellular localization assays. In brief, *A. tumefaciens* (GV3101) harboring the gene of interest on a binary plasmid was grown on a selective Luria broth (LB) medium. The bacteria were pelleted and resuspended in water. The plants were then infiltrated with *A. tumefaciens* suspensions at an optical density (OD) of 0.1–0.4 at 600 nm by injecting the bacteria into the abaxial side of the leaf using a syringe without a needle.

In all cases, the construct that expressed the silencing suppressor p19 (pBin61-P19) (59) was coinfiltrated at an OD_600_ of 0.25. After 3 days, the leaves were analyzed on a confocal laser scanning microscopy Leica TCS SP5 II (LAS AF program was used for capturing images) with an HCX PL APO CS 63.0× 1.40 UV oil immersion objectives or a Zeiss 780 Confocal microscope (Zen 2011 program was used for capturing images) with a 40× water immersion objective. The detectors used were two PMT detectors and a GaAsP detector of high sensitivity (32 channels) (which allows working in the photon-counting mode). Excitation/emission wavelengths were 488/524–550 nm for eGFP, 433/445–475/503 nm for CFP, 514–527 nm for YFP, and 543/566–634 nm for mRFP. Images were processed with ImageJ software. Percentage of colocalization was calculated as the ratio of yellow pixels (colocalization areas) to the total area of the organelle/substructure using threshold color tool (ImageJ) in microscope figures. Pearson correlation coefficient (Pearson’s *Rr*) was calculated using colocalization finder tool of ImageJ.

For the BiFC experiments, culture mixtures of *A. tumefaciens* carrying the different BiFC plasmids (OD_600_ = 0.3) were coagroinfiltrated in *N. benthamiana* plants with the vector Fibrillarin-mRFP (OD_600_ = 0.1) (60) and the vector containing the p19 suppressor protein (OD_600_ = 0.2) (59). Transfected cells expressing each BiFC pair were microscopically analyzed 3–4 days post-agroinfiltration (dpa).

### Nucleus enrichment from *N. benthamiana* tissue

Leaves expressing the tested proteins (5 g) were ground in liquid nitrogen until obtaining a fine powder, then resuspended in 40 mL of extraction buffer I (20 mM Tris-HCl [pH 8], 0.4 M sucrose, 10 mM MgCl_2_, 5 mM β-mercaptoethanol, and 0.2 mM PMSF) and kept on ice for 10 min, with inversion mixing every 2 min. The plant extracts were filtered using Whatman filter paper to remove solid plant material. The samples were centrifuged at 2,000 × *g* and 4°C for 20 min. Each pellet was resuspended in 20 mL of extraction buffer II (10 mM Tris-HCl [pH 8], 0.25 M sucrose, 10 mM MgCl_2_, 5 mM β-mercaptoethanol, 1% Triton X-100, and 0.2 mM PMSF) and centrifuged at 2,000 × *g* and 4°C for 10 min. The pellets containing nuclei were gently resuspended in 500 µL of extraction buffer III (10 mM Tris-HCl [pH 8], 1.7 M sucrose, 2 mM MgCl_2_, 5 mM β-mercaptoethanol, 0.15% Triton X-100, 0.2 mM PMSF), and then another 500 µL of the extraction buffer III were added gently. The processed pellets were centrifuged for 10 min at 13,000 rpm and 4°C to precipitate the nuclei.

### Co-immunoprecipitation

The proteins were extracted after performing nuclei enrichment by adding 300 µL of RIPA buffer (10 mM Tris-HCl [pH 7.5], 150 mM NaCl, 0.5 mM EDTA, 0.1% SDS, 1% Triton X-100, 1% sodium deoxycholate, 2.5 mM MgCl_2_, and 1 mM PMSF). The samples were sonicated with rounds of 30 s × 30 s between sonication for 30 min (TESTLAB Ultrasonic Cleaner; 260 W power and 40 kHz frequency). The samples were kept on ice throughout the procedure, and then centrifuged for 10 min at 10,000 rpm and 4°C. The supernatant was incubated overnight with 25 µL of GFP-Trap (Chromotek, Germany) at 4°C with continuous shaking. Then they were centrifuged at 2,500 × *g* for 2 min at 4°C. The supernatant was discarded and the beads were washed three times with dilution buffer (10 mM Tris-HCl [pH 7.5], 0.5 mM EDTA, and 150 mM NaCl). Subsequently, 30 µL of RIPA buffer and 15 µL of SB3X buffer (0.15 M Tris-HCl [pH 6.8], 6% SDS, 30% glycerol, 30% β-mercaptoethanol, and 0.075% bromophenol blue in 8 M urea) were added to the processed pellets. Finally, 0.36 µL of 0.5 M DTT was added to each sample. The samples were heated at 105°C for 15 min, and subsequently centrifuged at 2,000 × *g* for 2 min before being used and loaded on the gel.

### Protein analysis

Four leaf discs (0.8 cm in diameter) were excised from *N. benthamiana* leaves expressing the desired proteins and ground in liquid nitrogen until obtaining a fine powder. The obtained powder was resuspended in 200 µL of the protein extraction buffer (75 mM Tris-HCl [pH 6.8], 30% glycerol, 5% β-mercaptoethanol, and 2% SDS). This extract was centrifuged at 13,000 rpm for 2 min, and the supernatant was used for immunoblot analysis by adding 200 µL of 4× SB (62.5 mM Tris-HCl [pH 6.8], 2% SDS; 10% glycerol, 5% β-mercaptoethanol, and 0.001% bromophenol blue). The samples were boiled for 5 min, and centrifuged for 2 min at 13,000 rpm.

The clarified supernatants were separated on 12% SDS-PAGE. Then, the proteins of the gels were transferred to PVDF membranes (Amersham Hybond-P; GE Healthcare), and blocked with 5% non-fat milk powder in Tris-buffered saline containing 0.05% Tween-20. GFP (or its variants, CFP or YFP) and RFP fusion proteins were detected with anti-GFP (3H9) monoclonal antibody (Chromotek, Germany) and anti-RFP (6G6) monoclonal antibody (Chromotek, Germany), respectively. Goat anti-rat IgG (Biosystems, BA, Argentina) and anti-mouse IgG (GenScript, NJ, USA) HRP-conjugated antibodies were used as secondary antibodies. A chemiluminescent reagent was used to detect the peroxidase activity, according to the manufacturer’s instructions (ECL Plus Western blotting detection reagents; GE, UK). An anti-HYL1 antibody (Agrisera) and goat polyclonal anti-rabbit IgG-HRP secondary conjugated antibody (Agrisera) were used in co-immunoprecipitation (co-IP) assays with HYL1 and 24K.

### Protein bioinformatics analyses

The 24K protein sequence used for the bioinformatics analyses was GenBank AAO34633.1. Nuclear localization signals (NLSs) and nucleolar localization signals (NoLSs) were searched using ScanProsite (61), WoLF PSORT (62), and NOD (63, 64). NetNES (65) was used to predict nuclear export signal (NES) and FastRNABindR (66) and Pprint (67), to identify RNA binding sites. Agos server (68) and I-TASSER (69) were used to identify WG/GW motifs and to obtain 3D structure of 24K protein, respectively. Verify_3D (70–72) and ProSA (73) were used to evaluate 3D results. The hydrophobicity was analyzed using the Kyte and Doolittle scale with ProtScale (74). The F-box motif (IPR001810) was predicted using InterPro (75) database and filtering *C. sinensis*. Alignment analyses included 328 *C*. *sinensis* protein sequences and the 24K sequence. A logo was generated using WebLogo server (76).

## RESULTS

### CPsV infection alters miRNA biogenesis leading to an increment of unprocessed miRNA precursors in *C. sinensis* plants

**Fig 1:**
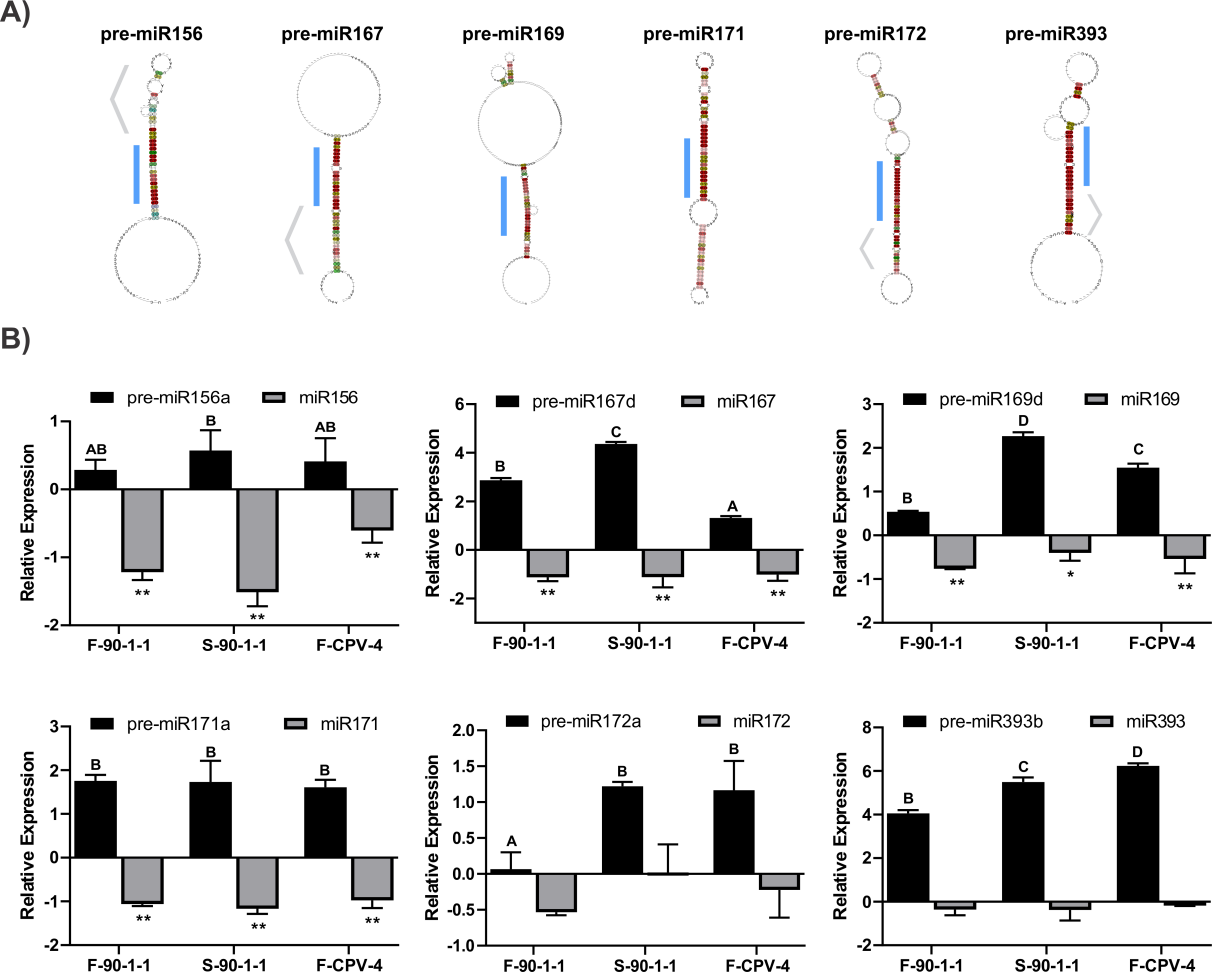
Analysis of relative accumulation of miRNA precursors and mature species in *C. sinensis* plants infected with two CPsV isolates (90-1-1 and CPV-4). (**A**) Representation of the consensus secondary structure obtained with RNAalifold for each family of pre-miRNA analyzed. The light blue bar indicates the position of the miRNA/miRNA* duplex. Regarding the pre-miR156 family, the upper terminal loop (indicated by a gray arrowhead) shows a conserved pattern associated with the loop to base processing model. Pre-miR167, pre-miR172 and pre-miR393 families show higher conservation in the lower stem regions associated with a base to loop processing model (indicated by a gray arrowhead in each case). For the pre-miR171 and pre-miR169 families, the consensus secondary structure reflects base to loop and loop to base processing determinants coming from different individual pre-miRNAs inside each family. (**B**) Black bars: quantitative reverse transcription–polymerase chain reaction (RT–qPCR) assays in sweet orange plants were performed to determine the accumulation of the following precursors: miR156*a*, miR167*d*, miR169*d,* miR171*a,* miR172*a*, and miR393*b*. The numbers were calculated as log2 of the infected/healthy sample ratios (healthy = 0). The different letters show significant differences using a one-way ANOVA (Tukey *post hoc* test; *P* < 0.05). Healthy samples belong to statistically group (**A**) Gray bars: relative accumulation of mature miRNA species. The numbers were calculated as log2 of the infected/healthy sample ratios (healthy = 0). Differences between infected and healthy groups were tested with a one-way ANOVA (Tukey *post hoc* test) * and ** indicate *P* < 0.05 and *P* < 0.01 values, respectively. Mean values and standard errors of at least three independent experiments are shown.

We have previously reported that the infection of sweet orange plants with two isolates of CPsV expressing different symptomatology leads to the downregulation of mature species of some endogenous miRNAs (45), with a concomitant upregulation of some of their mRNA targets (51). In the present study, we used publicly available data (77, 78) to obtain the sequences of *C. sinensis* pre-miRNAs corresponding to the studied downregulated mature species, and then performed a prediction of the pre-miRNA folding structures using different members of each family and compared their conservation with those of *A. thaliana* (Fig. 1A).

The observed conservation between structures suggests that the direction of the processing (loop to base or base to loop 79, 80]) is also conserved regarding those described for *A. thaliana* (Fig. 1A). We then used transcriptomic data to identify pre-miRNA members of selected families with the highest accumulation levels (miR156*a*, miR167*d*, miR169*d*, miR171*a*, miR172*a*, and miR393*b*) (77, 78).

To evaluate if an alteration in the processing of miRNA precursors could explain the downregulation of the mature miRNAs in the virus-infected samples, we quantified the accumulation of the selected precursors by RT-qPCR in *C. sinensis* inoculated with two CPsV isolates (90-1-1 or CPV-4) presenting characteristic symptoms (flecking and *shock* for CPsV 90-1-1 and only flecking for CPV4). Healthy plants challenged with healthy tissue were also analyzed as controls.

The miR156*a* precursor showed an incremented level in the infected plants compared to the healthy samples (shock symptoms (S-90-1-1) had the highest increment with a 0.57-fold upregulation expressed as log2 of the infected/healthy sample), similar to reported in our previous research (45). The miR167*d* precursor showed an increase of 2.86-fold for the sample F-90-1-1, and a much greater upregulation for the S-90-1-1 sample (4.36-fold higher than those of the uninfected control; Fig. 1B). Reduced accumulations of mature miRNA species were also evident for the same samples (45). This finding confirms the opposite behavior for precursor accumulation. In the case of the miR169*d* precursor, the upregulation levels were 0.54- and 1.55-fold for F-90-1-1 and F-CVP-4 respectively, whereas S-90-1-1 exhibited an increase of 2.27-fold (Fig. 1B).

The miR171*a* precursor showed a similar behavior between the two isolates evaluated and the symptoms observed (flecking and shock). All samples showed a significant increase with respect to the healthy control. The levels were 1.76-, 1.73-, and 1.61-fold higher in F-90-1-1, S-90-1-1, and F-CPV-4 infected samples, respectively (Fig. 1B).

The miR172*a* levels showed significant differences for S-90-1-1- and F-CPV-4-infected samples, with an increase of 1.22- and 1.17-fold, respectively.

The miR393*b* precursor showed the most drastic alterations (4.05- and 5.50-fold higher in F-90-1-1- and S-90-1-1-infected samples, respectively). Besides, in the samples infected with the CPV-4 isolate (6.23), miR393*b* was the only precursor with levels exceeding those of the S-90-1-1 samples (Fig. 1B).

Altogether, the results revealed a higher accumulation of the unprocessed species in infected samples for the six evaluated precursors, which correlates with the downregulation of the mature miRNA species (45). Moreover, the increased accumulation of the miRNA precursors correlated with the severity of the symptoms, that is, the samples with shock showed the greatest changes compared to the healthy samples. Besides, the levels of the primRIPary transcript in the infected leaves showed no alterations compared with the healthy samples. This finding suggests that there is no higher induction of transcription (45).

**Fig 2:**
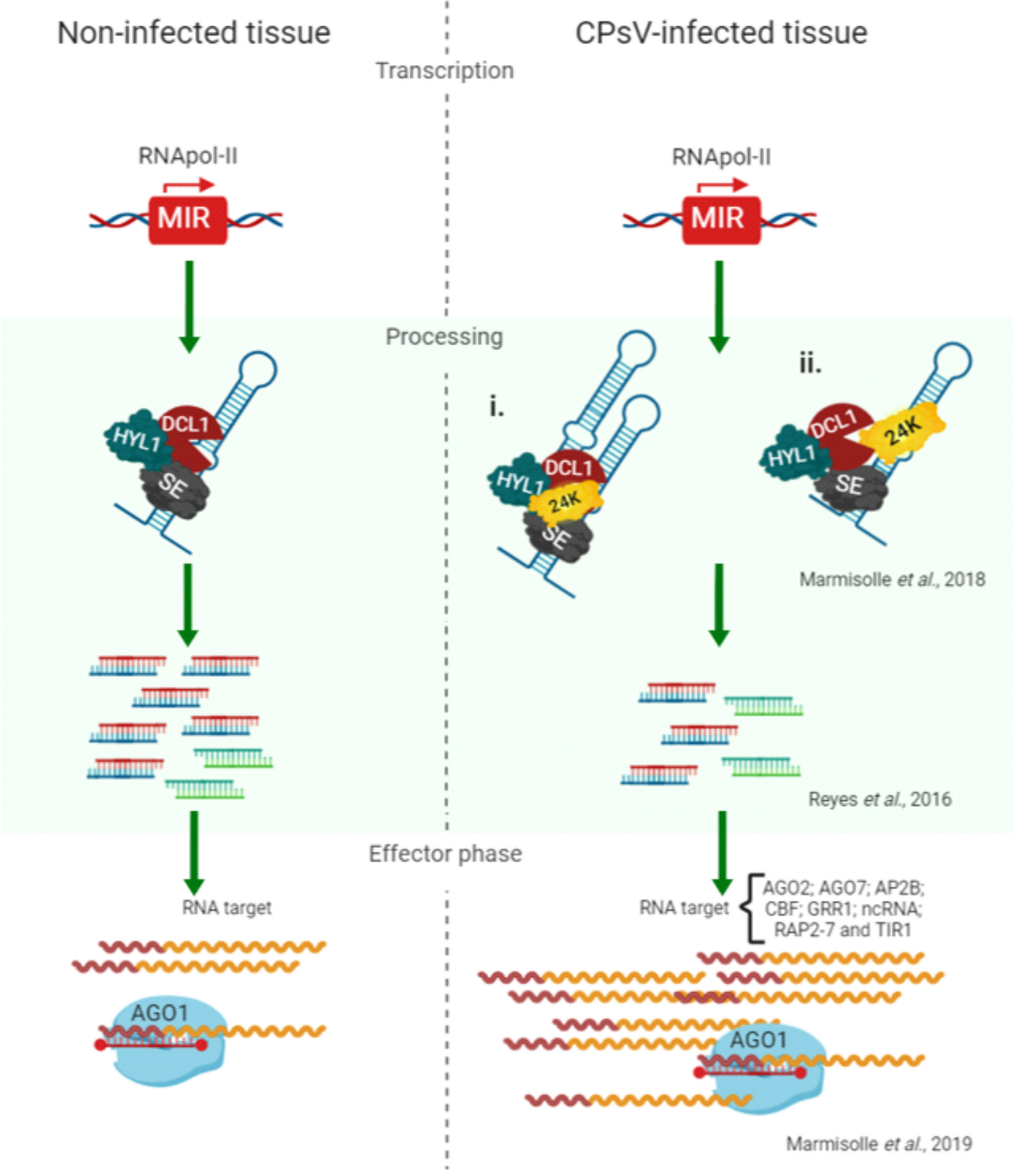
Proposed model of interference in biogenesis and effector phase of miRNA-mediated silencing caused by 24K protein. The left part represents conditions of non-infected tissue, where MIR genes are transcribed and normally processed by DCL1 and its accessory proteins HYL1 and SE to release a basal amount of mature duplex miRNA/miRNA*. Once in the cytoplasm, RISC recognizes target transcripts to carry out its negative regulatory effects. The right part represented the proposed scenario for a CPsV-infected plant cell. After MIR genes are transcribed, 24K would interact with pre-miRNAs (45) and with accessory proteins of the miRNA biogenesis (HYL1 and SE; shown here). These interactions would alter pre-miRNA processing, which would result in lower miRNA/miRNA* accumulation (45). Duplexes load onto AGO1 form RISC silencing complexes but will lead to a decrease in target degradation (higher accumulation of targets, including CCAAT-binding transcription factor family [CBFAs]; ethylene-responsive transcription factor [RAP2-7]; integrase-type DNA-binding superfamily protein [AP2B], and so on; [51]).

Interestingly, a reduction of mature miRNA accumulation with an overaccumulation of unprocessed precursors is a common feature in plants defective in miRNA processing, such as *hyl1* or *dcl1* mutants (15, 81, 82). This parallelism, together with the finding that 24K protein interacts with precursors in *in vivo* RNA immunoprecipitation assays (RIP) (45), led us propose a model in which the CPsV 24K protein targets the miRNA biogenesis complex (Fig. 2). This targeting could occur through either RNA binding (i) or the interaction with protein components of the processing machinery (ii) (51). This would finally lead to a reduction in mature miRNA species and an increment in specific target transcripts such as CBFs (CCAAT-binding transcription factor subunit B), ethylene-responsive transcription factor (RAP2-7) or integrase-type DNA-binding superfamily protein (AP2B) (51).

### 24K gene encodes a nuclear protein with recognizable RNA and protein interacting domains

The viral protein versatility allows the virus to accomplish all the necessary functions, such as replication and dissemination, using only a few proteins (83–85). One of these proteins, 24K, binds long dsRNA *in vitro* (86) and miRNA precursors *in vivo* (45). Those abilities would account for the misprocessing and overaccumulation of miRNA precursors.

**Fig 3:**
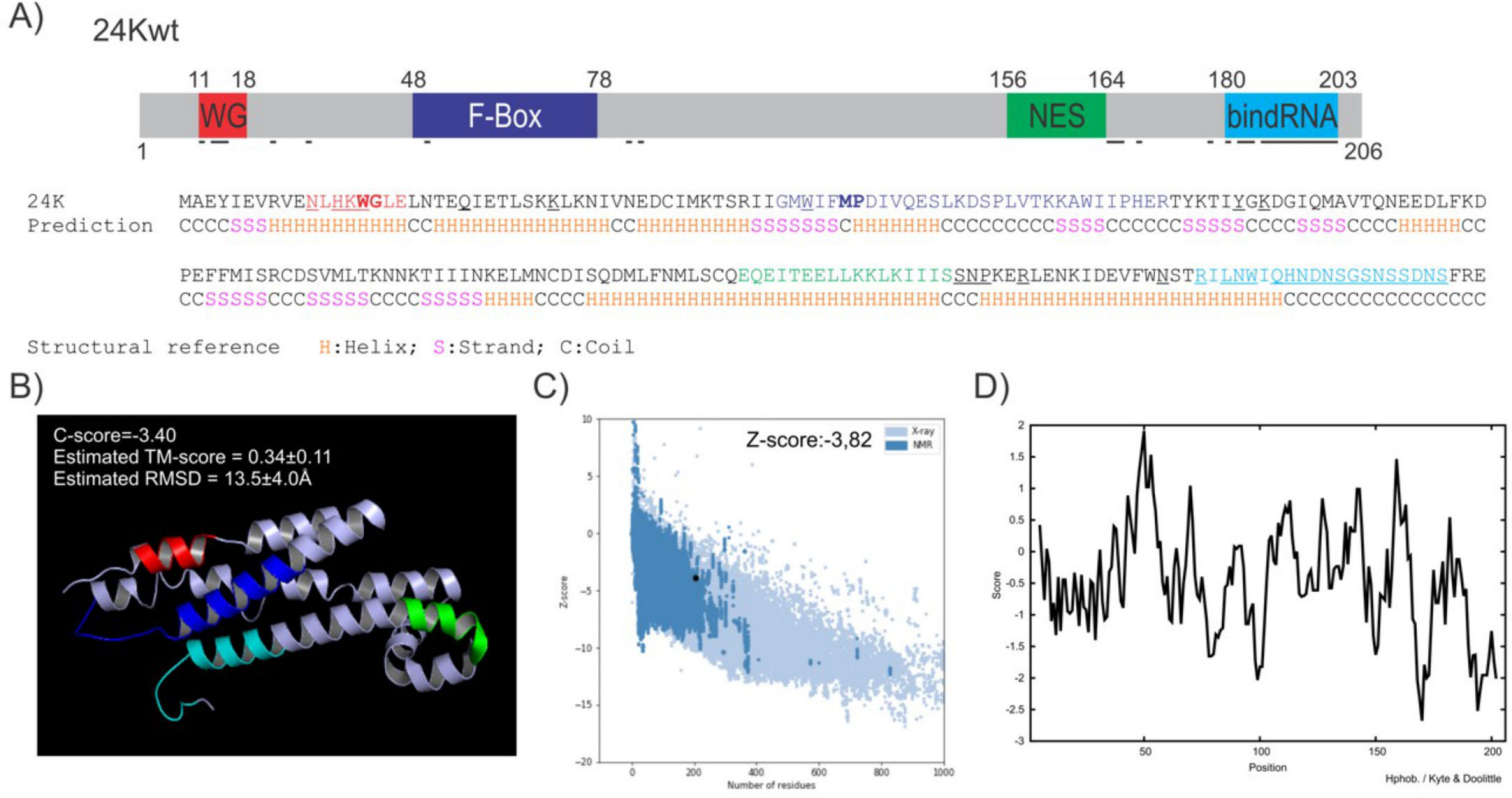
Bionformatic analysis of the 24K protein. (**A**) (i) Schematic representation of predicted motifs (WG/GW, F-box, NES, and RNA binding) present in the viral protein. (ii) Prediction of secondary structure of 24K by I-TASSER. (H: alpha helix, C: coil, S: beta strand). Amino acid colors (red, blue green and light blue) refer to the identified motifs (i). (**B**) Predicted 3D structure of 24K by I-TASSER. The selected model has a C-score of −3.40 and uses as template a crystalized *Saccharomyces cerevisiae* protein (PDB: 1FIO_A). (**C**) The 3D-model quality was verified by ProSa with a *Z*-score of −3.82. (**D**) The hydrophobicity profile was analyzed by ProScale for each residue position using the scale of Kyte and Doolittle (87).

With this in mind, an *in silico* prediction of the RNA binding sites in the 24K protein sequence performed using FastRNABindR (66) and Pprint (67) revealed two specific regions: one comprising N^11^, H^13^, K^14^, and W^15^ and a C-terminal region (^180^NSTRILNWIQHNDNSRSNSSDNS^203^) (Fig. 3A).

Protein-protein interaction may also be responsible for miRNA misprocessing. WG/GW motifs (88) are recognized as essential for AGO interaction and RNA silencing throughout eukaryote kingdoms (89–96). GW motifs are also involved in nucleolar localization and sRNA binding capability (97). An analysis of WG/GW motifs in 24K using Agos server (68) predicted the presence of a putative motif (^11^NLHKWGLE^18^) but with low compositional compatibility (Fig. 3A).

Researchers have also described F-box motifs as responsible for the interaction between viral proteins and plant-silencing machinery (30, 98–100). Local alignments between the 24K amino acid sequence and *C. sinensis* F-box proteins (FBPs) allowed the identification of a hydrophobic region comprising 30 amino acids between the amino acids 48 and 78 in the 24K sequence (rich in V, I, M, L, and P). The F-box conserved MP dipeptide at the 3′ of the motif was also present (Fig. 3A).

Finally, we created a 3D-structure model of 24K using I-TASSER (69) to visualize and locate the identified regions and motifs (Fig. 3B). The model quality was validated using Verify_3D (70–72) and ProSA (73) (Fig. 3C). A hydrophobicity profile analyzed using ProScale (76; Fig. 3D) revealed two hydrophobic regions. The hydrophobic region with the higher score was between the 47 and 55 positions and included the F-box. The other located inside a predicted NES (positions 158–163). The segment around position 50 could be indicative of a protein-protein interaction region.

### 24K protein forms aggregates in the nucleus and nucleolus and colocalizes with fundamental components of the miRNA biogenesis machinery

**Fig 4:**
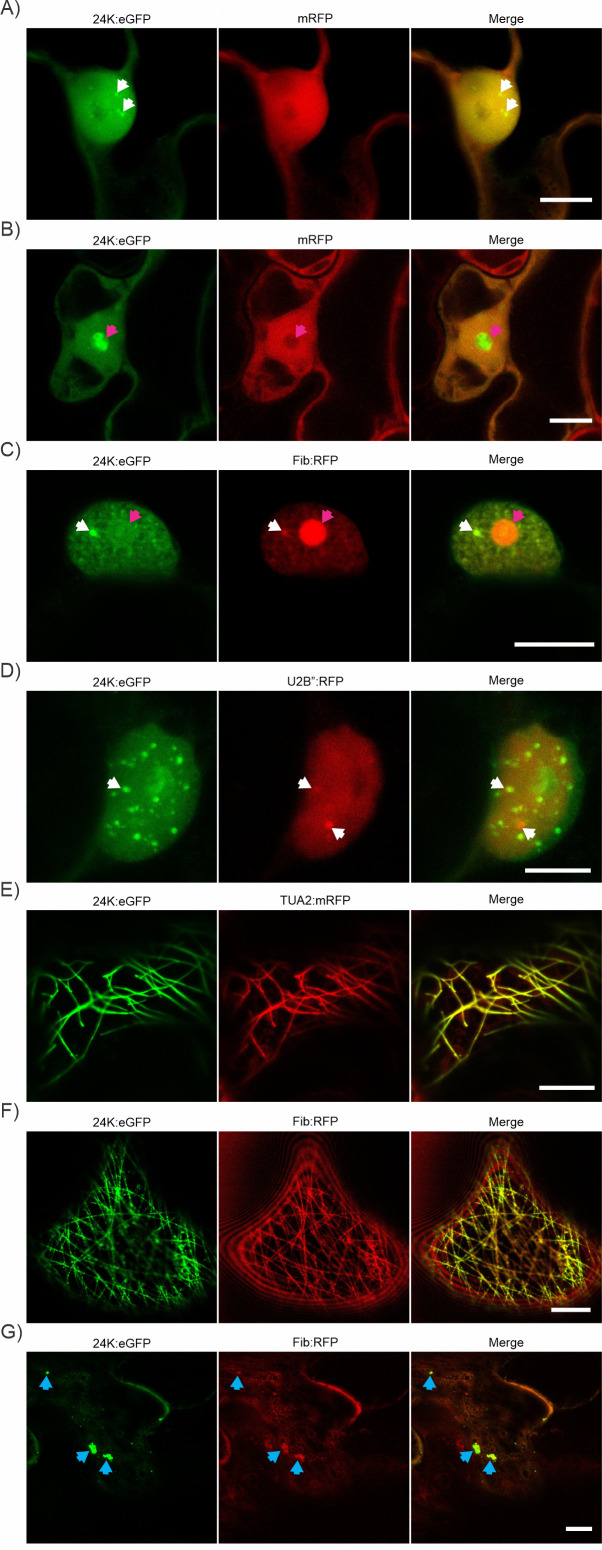
Subcellular localization of 24K. (**A and B**) Coexpression of 24K:eGFP with mRFP. (**A**) Localization of 24K:eGFP in nucleus aggregates (white arrowheads). (**B**) 24K:eGFP occupation of the nucleolus (pink arrowheads). (**C**) Colocalization of 24K:eGFP with Fib:RFP in nucleoli and in certain nucleoplasm aggregates (pink arrowheads). (**D**) 24K:eGFP partially colocalizes with U2B″:RFP aggregates (white arrowheads). (**E**) Colocalization of 24K:eGFP with TUA2:mRFP in microtubules. (**F and G**) Relocalization of Fib:RFP to microtubules and cytoplasm aggregates (blue arrowheads) by 24K:eGFP, respectively. Scale bar: 10 µm.

Transient expression of 24K fused to reporter proteins revealed nuclear and nucleolar localization in *N. benthamiana* epidermal cells. This protein forms aggregates in the nucleoplasm (Fig. 4A, C and D) and also accumulates in the cytoplasm (Fig. 4E, F and G) (45, 101). It aggregated and partially colocalized with the nucleolar and Cajal bodies (CBs) marker Fibrillarin (Fib) and the specific CBs marker U2B protein (U2 small nuclear ribonucleoprotein B) (60) (Fig. 4C and D). Thus, 24K could accumulate also in other types of aggregates, such as D-bodies (102). Localization quantification was performed in different organelles or substructures (Table 2), confirming a high level of localization of the 24K protein in the nucleoplasm and very low localization in CBs.

**TABLE 2:** Colocalization quantification between 24K:eGFP and different specific organelle markers^*a*^

	24K:eGFP	
	Percentage of colocalization in the organelle/substructure	Pearson’s *Rr* (overlap *R*)
mRFP_Nucleoplasm_	65.80	0.16 (0.99)
Fib:RFP_Cajal bodies_	30.29	−0.61 (0.95)
U2B:RFP_Cajal bodies_	5.74	0.10 (0.99)
TUA2:RFP_Microtubules_	52.67	0.32 (0.98)
Fib:RFP_Fibrillarin networks_	18.25	0.15 (0.98)
Fib:RFP_Cytoplasm aggregates_	1.58	0.15 (0.98)

^*a*^ Colocalization was quantified by two methods: percentage of colocalization and Pearson correlation coefficient. Percentage of colocalization was calculated as the ratio of yellow pixels (colocalization areas) to the total area of the organelle/sub-structure using threshold color tool (ImageJ) in microscope figures (merge panels). Pearson correlation coefficient (Pearson’s *Rr*) was calculated using colocalization finder tool of ImageJ. Values ranged from −1 and 1 for different degrees of colocalization (−1: no colocalization; 1: 100% colocalization). Overlap *R* is the statistical interpretation of the Pearson’s *Rr.*

Besides, the 24K viral protein formed cytoplasmic filaments in cortical region of *N. benthamiana* cells. The co-expression of 24K with TUA2:mRFP, a microtubule marker (103), revealed association of the viral protein with microtubules (Fig. 4E). The colocalization with Fib:RFP in cytoplasmic aggregates and microtubules suggests a possible 24K-mediated relocation of Fib:RFP from nucleolus to the cytoplasm (Fig. 4F and G), as reported for the ORF3 protein from CMV (104, 105). Colocalization quantification was performed in different cytoplasmic substructures (Table 2), confirming higher localization of the 24K protein in microtubules and moderate colocalization in Fib networks and cytoplasmic aggregates.

Fang and Spector (102) showed that reporter-fused proteins from the miRNA biogenesis pathway, CFP:AtDCL1 and AtHYL1:YFP, colocalize in discrete round nuclear bodies in *A. thaliana* and that a high number of them are close, but not within, nucleoli. However, AtSE:YFP was distributed in a heterogeneous sub-nuclear pattern forming speckles or interchromatin granules (102). Here, subcellular colocalization assays performed by transient expression of CFP:AtDCL1, AtHYL1:YFP or AtSE:YFP and 24K:mRFP in *N. benthamiana* leaves revealed that 24K colocalizes in aggregates different from the CBs, as evidenced by confocal microscopy. Colocalization quantification was performed between 24K:mRFP and CFP:AtDCL1, AtHYL1:YFP, or AtSE:YFP (Table 3). Although 24K:mRFP colocalized with all three miRNA machinery proteins, the percentage of colocalization, considering the complete nucleus area, was 2.5 times higher between 24K:mRFP and AtHYL1:YFP than between 24K:mRFP and AtSE:YFP. The percentage of colocalization between 24K:mRFP and CFP:AtDCL1, in the total nucleus area was the lowest.

**TABLE 3:** Colocalization quantification between 24K:eGFP and different miRNA biogenesis proteins (CFP:AtDCL1, AtHYL1:YFP, and AtSE:YFP)^*a*^

	Percentage of colocalization in nuclear aggregates	Pearson’s *Rr* (overlap *R*)	Percentage of colocalization in the nucleus
24K:mRFP			
CFP:AtDCL1	26.16	0.36 (0.99)	3.24
AtHYL1:YFP	46.73	0.14 (0.99)	23.59
AtSE:YFP	22.56	0.45 (0.99)	9.15
mRFP			
CFP:AtDCL1	9.42	−0.0 (NaN)	0.92
AtHYL1:YFP	8.32	−0.0 (NaN)	2.06
AtSE:YFP	8.50	−0.21 (0.99)	3.25

^*a*^ Colocalization was quantified by two methods: percentage of colocalization and Pearson correlation coefficient. Percentage of colocalization was calculated as the ratio of yellow pixels (colocalization areas) to the total area of the organelle/sub-structure using threshold color tool (ImageJ) in microscope figures (merge panels). Pearson correlation coefficient (Pearson’s *Rr*) was calculated using colocalization finder tool of ImageJ. Values ranged from −1 and 1 for different degrees of colocalization (−1: no colocalization; 1: 100% colocalization). Overlap *R* is the statistical interpretation of the Pearson’s *Rr.* NaN: not a number. Colocalization quantification between non-fused mRFP and miRNA biogenesis protein was also shown as control (lower table).

**Fig 5:**
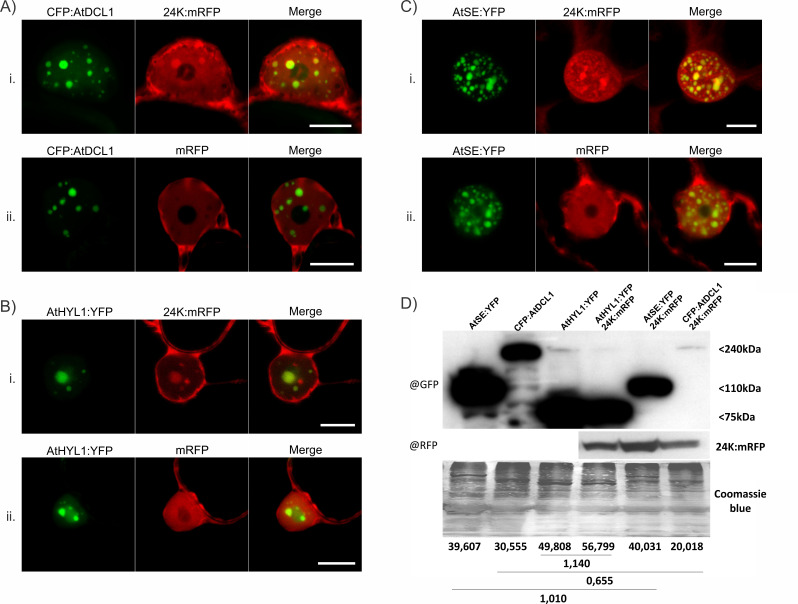
Subcellular colocalization of 24K and miRNA biogenesis proteins. (**A**) (i) Colocalization of 24K:mRFP with CFP:AtDCL1 in nuclear aggregates. (**B**) (i) Colocalization of 24K:mRFP with AtHYL1:YFP in nuclear aggregates. (**C**) (i) Colocalization of 24K:mRFP with AtSE:YFP in nuclear aggregates. (ii) The mRFP control does not colocalize in nuclear aggregates with any of the tested proteins. (**D**) Western blot analyses of extracts from *N. benthamiana* leaves in colocalization assays at 3 dpa. Anti-RFP (@RFP) or anti-GFP (@GFP) monoclonal antibodies were used for 24K or biogenesis proteins, respectively. Coomassie blue stain is shown as loading control. The numbers below correspond to normalized band density. Ratios between AtDCL and AtDCL1/24K (240 kDa); AtHYL1 and AtHYL1/24K (75 kDa); AtSE and AtSE/24K (110 kDa) are shown under the lines. Scale bar: 10 µm.

The distribution and quantity of aggregates observed corresponded to those formed by proteins involved in miRNA biogenesis. This finding would indicate 24K cospecifically localizes in D-bodies with the three proteins tested, DCL1 (Fig. 5A), HYL1 (Fig. 5B), and SE (Fig. 5C). On the other hand, the non-fused mRFP protein, used as a negative control, did not colocalized with these proteins, thus indicating that the observed pattern was dictated by the viral protein 24K and not by the fused reporter. Western blot analyses confirmed the expression of the assayed proteins (Fig. 5D) and an apparent destabilization of AtDCL1 when co-expressed with 24K.

### 24K protein interacts with HYL1 and SE

The nuclear colocalization of 24K with miRNA biogenesis proteins suggested that it may associate or interact with the nuclear processing complex or, at least, with some of their components. To explore this possibility, we tested the capacity of 24K to interact with AtDCL1, AtHYL1 or AtSE by BiFC and Co-IP assays. The fusion proteins used for the BiFC assays were AtDCL1:mCitrine (19), AtHYL1:mCitrine, and AtSE:mCitrine (58). Coexpression of N-mCitrine:AtDCL1, N-mCitrine:AtHYL1, or N-mCitrine:AtSE with C-mCitrine:24K in *N. benthamiana* leaves was performed. N-mCitrine and C-mCitrine empty vectors were used as negative control and Fib:mRFP as expression control.

**Fig 6:**
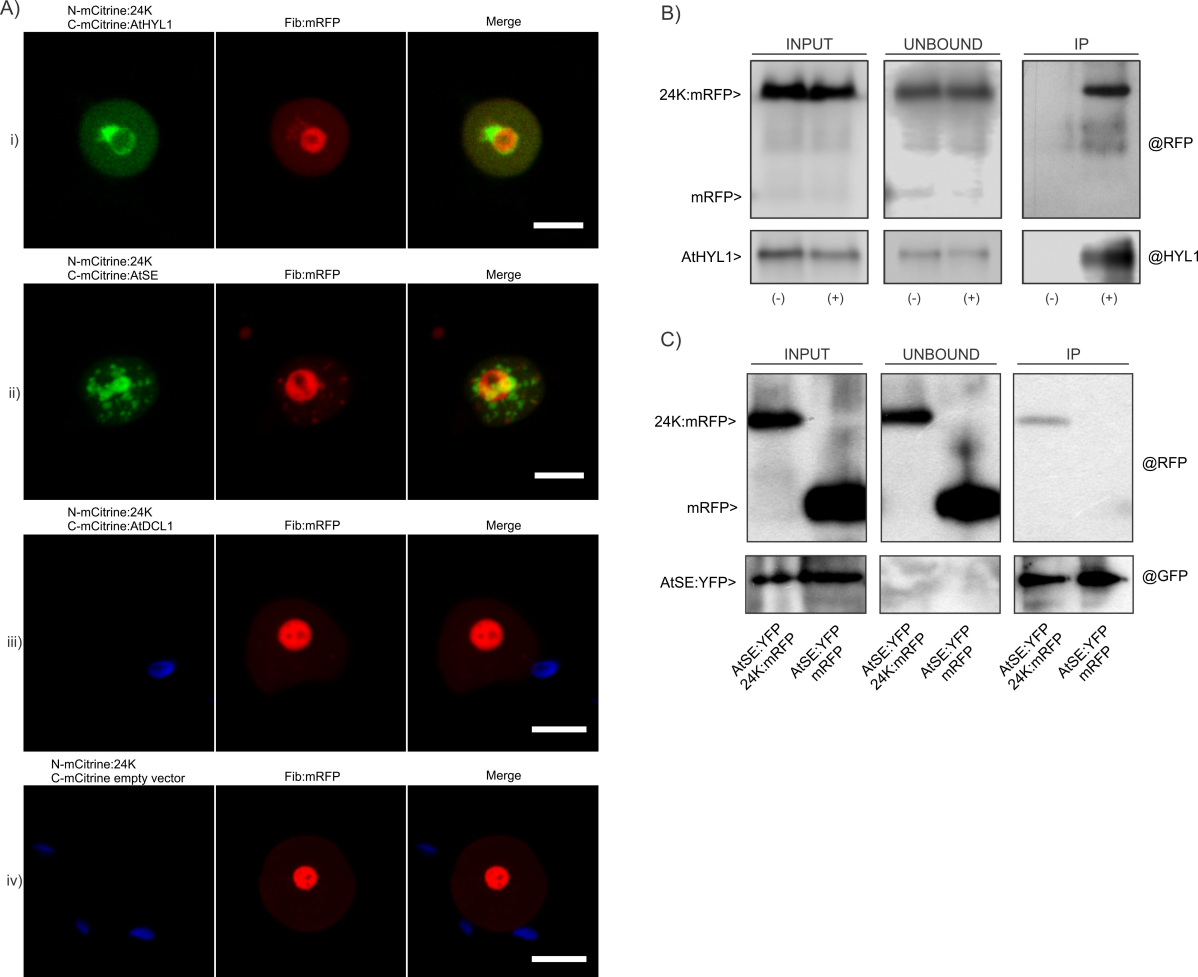
Interaction between 24K protein and miRNA biogenesis proteins. (**A**) *In vivo* analysis of BiFC assays in epidermal cells of *N. benthamiana* plants. The merge panels show positive interaction between N-mCitrine:24K and C-mCitrine:AtHYL1 (i) and N-mCitrine:24K and C-mCitrine:AtSE (ii) and no interaction between N-mCitrine:24K with C-mCitrine:AtDCL1 (iii). (iv). Negative control: N-mCitrine:24K + C-mCitrine (empty vector). Chloroplasts were marked in blue in the case of the negative interactions. In all cases, Fib:mRFP was used as an expression control of infiltrated samples. Scale bar: 10 µm. (**B**) Co-IP assay between 24K:mRFP and AtHYL1. INPUT and UNBOUND controls correspond to input and output fractions without binding to bead system, respectively. The IP fraction corresponds to immunoprecipitated proteins. (−) corresponds to the negative control using beads without @HYL1 antibody. (+) corresponds to the immunoprecipitated samples with @HYL1 attached to the beads. The anti-RFP (@RFP) and anti-HYL1 (@HYL1) monoclonal antibodies were used in each case to develop the blots. The presence of both proteins in the IP fraction indicates a positive interaction. (**C**) Co-IP assay between the 24K:mRFP and AtSE:YFP protein. The INPUT and UNBOUND controls correspond to input and output fractions without binding to bead system, respectively. The IP fraction corresponds to immunoprecipitated proteins with @GFP attached to the beads. Free mRFP was used as a negative control. The anti-RFP (@RFP) and anti-GFP (@GFP) antibodies were used in each case to develop the blots. The presence of both proteins in IP fraction indicates a positive interaction.

Confocal images revealed that C-mCitrine:24K associates with N-mCitrine:AtHYL1 and N-mCitrine:AtSE but not with N-mCitrine:AtDCL1 (Fig. 6A). A pattern of a ring-shape large aggregate in contact with the nucleolus was observed in the case of 24K-AtHYL1 interaction similar to that previously described by Han et al. (81), for AtHYL1 localization (Fig. 6Ai). This interaction was not exclusively in the nuclear bodies, but also occurred in the cytoplasm. In the case of the 24K-AtSE interaction, a high number of nuclear aggregates were evident (Fig. 6Aii).

We also confirmed the capacity of 24K to interact with AtHYL1 by Co-IP using an mRFP-tagged version of the viral protein and an antibody against AtHYL1 (Fig. 6B) in transitory expression experiments in *N. benthamiana* leaves. On the other hand, the analysis of the possible interaction of AtDCL1 and AtSE with 24K using tagged versions of the three proteins (CFP:AtDCL1, AtSE:YFP, and 24K:mRFP) demonstrated that only AtSE (Fig. 6C) interacted with 24K in *N. benthamiana* leaves. These results perfectly correlated with BiFC observations.

With the objective of identifying regions in 24K involved in the interaction with miRNA biogenesis proteins, a series of four mutants was assayed (101). 24KΔN lacks the first 33 amino acids from N-terminal end, possibly involved in the subcellular targeting of the protein. 24KW^15^A has a specific substitution of Trp-15 by Ala (corresponding to the WG motif). In the case of 24KΔNES, a part of the nuclear exportation signal, was deleted. Finally, 24KΔC mutant lacks amino acids comprising a putative RNA binding domain. All mutants were tested for possible interaction with similar versions of the proteins involved in miRNA biogenesis. All evaluated mutants maintained the interaction with AtHYL1 or AtSE with localization patterns similar to that of the wild-type (wt) 24K (web resource: https://figshare.com/articles/figure/Figure_Mutants_pdf/25565163). Moreover, all mutants showed no interaction with AtDCL1 as occurred with 24Kwt.

## DISCUSSION

In a previous study, we analyzed the accumulation of several conserved mature miRNAs in citrus plants infected with two distantly related CPsV isolates that induce symptoms of different severity (45). We showed that viral infection reduces mature species accumulation of several endogenous miRNAs probably by interference of one or more of the viral proteins with some components of the miRNA biogenesis pathway.

In the present study, CPsV infection also increased unprocessed miRNA precursors in *C. sinensis* plants (Fig. 1), thus confirming miRNA biogenesis misregulation by the virus. Two main hypotheses emerge to explain the processing alterations: (i) the viral protein can bind miRNA precursors impeding its processing or (ii) the viral proteins can alter protein components of the miRNA processing machinery (Fig. 2).

The 24K protein exhibited high affinity for long dsRNAs but not for small dsRNAs *in vitro* (86). The predicted RNA-binding domain in the C-terminal region of 24K (Fig. 3A) could explain the dsRNA capacity shown in *in vitro* and *in vivo*, including pre-miRNA binding (45, 86). The nuclear colocalization of 24K with the three main proteins of the miRNA processing complex (DCL1, HYL1, and SE) in round nuclear bodies similar to the reported D-bodies (Fig. 5) (102) is the first evidence suggesting an interaction between this viral protein and some component of the processing machinery. The presence of a putative F-box within a hydrophobic region of the protein and a WG/WG motif suggests a possible site of interaction with other proteins (Fig. 3A and D).

The processing of the pre-miRNAs occurs cotranscriptionally after an initial recruitment of factors (11); which suggests that the D-bodies might be associated with chromatin. Speth et al. (106) have shown that SE also binds to some MIR loci opening the possibility that SE binds to chromatin and phase separates to concentrate other D-body components for miRNA processing. A recent study demonstrates that SE forms droplets and drives DCL1, HYL1, and pri/pre-miRNAs into these D-bodies (107). D-bodies would be formed through SE-mediated phase separation and pre-miRNAs would be processed into mature miRNA duplexes in the droplets. After processing, mature miRNAs would bind to HYL1 and release D-bodies. Besides, HYL1–SE interaction and incorporation of HYL1 into SE droplets are enhanced by the presence of miRNA precursors (107). All these complex processing dynamics would explain the fact that 24K only interacts with HYL1 and SE in Co-IP and BiFC assays (Fig. 6), but not with DCL1, supported also by a lower colocalization of 24K with this protein (Table 3). The 24K interaction with miRNA precursors would also play a role in the association with SE and HYL1.

The NS3 protein from the rice stripe virus is a regulator of the processing of miRNA precursors that accelerates the process and increases the accumulation of mature miRNA species (42). A transgenic rice line overexpressing NS3 showed a reduction in a group of miRNA precursors; thus, this viral protein would promote the recruitment of precursors by the processing complex through the interaction with HYL1 (42). However, in contrast to NS3, 24K produces a decrease in mature miRNAs species, mainly by disturbing the processing. NS3 binds to precursors and interacts with HYL1 facilitating its dimerization and regulating its interaction with pri-miRNAs (42). Conversely, 24K may prevent HYL1 dimerization and may even sequester miRNA precursors.

Particular miRNAs showed different degrees of dependence with HYL1 (108). HYL1 mutant plants caused greater changes in the accumulation of some miRNAs, but not others, which were associated sometimes to precursor structures (81, 82, 109–112). The CPsV infection induces a downregulation of most of the tested endogenous miRNAs (7/10) in *C. sinensis* (45). The 24K protein interacted with HYL1 in the nucleus (Fig. 6). This protein may be responsible for altering the functioning of HYL1, thus causing the defective processing of a group of miRNAs more dependent on it. The interaction with HYL1 was also evident at the cytoplasmic level. This finding suggests that 24K may hijack HYL1 causing a reduction of its nuclear accumulation.
